# Design Development and Analysis of a Partially Superconducting Axial Flux Motor Using YBCO Bulks

**DOI:** 10.3390/ma14154295

**Published:** 2021-07-31

**Authors:** Brahim Chelarem Douma, Bilal Abderezzak, Elhadj Ailam, Raluca-Andreea Felseghi, Constantin Filote, Catalin Dumitrescu, Maria Simona Raboaca

**Affiliations:** 1 Laboratoire de l’Énergie et des Systèmes Intelligent (LESI), University of Khemis Miliana, Road of Theniet el Had, Khemis Miliana 44225, Algeria; b.chelarem-douma@univ-dbkm.dz (B.C.D.); b.abderezzak@univ-dbkm.dz (B.A.); elhadj.ailam@univ-dbkm.dz (E.A.); 2 Faculty of Electrical Engineering and Computer Science, Ștefan cel Mare University of Suceava, Universităţii Street, No.13, 720229 Suceava, Romania; andreea.felseghi@ccm.utcluj.ro (R.-A.F.); simona.raboaca@icsi.ro (M.S.R.); 3 National Department Telematics and Electronics for Transports, University “Politehnica” of Bucharest, 060042 Bucharest, Romania; catalin.dumitrescu@upb.ro; 4 National Research and Development Institute for Cryogenic and Isotopic Technologies—ICSI Rm. Valcea, Uzinei Street, No. 4, P.O. Box 7 Raureni, 240050 Valcea, Romania

**Keywords:** axial flux motor, superconducting motor, superconducting bulk, magnetic field concentration, FEA simulation

## Abstract

In this work, authors have designed, constructed and tested a new kind of partially superconducting axial flux machine. This model is based on the magnetic flux concentration principle. The magnetic field creation part consists of the NbTi superconducting solenoid and two YBaCuO plates. A theoretical study is conducted of an extrapolated superconducting inductor for low-temperature superconducting and high-temperature superconducting solenoids. The optimization of the inductor is carried out in order to increase the torque and the power density as well. This improvement is done by changing the shape of the elements which form the superconducting inductor. Finally, a prototype is realized, and tested.

## 1. Introduction

A very high power density in electrical machines can be obtained thanks to unconventional design of electromechanical converters. It is based on very high torque-to-weight and torque-to-volume ratios. The conventional electrical machines based on copper, iron and permanent magnets cannot deliver high power density, due to their inherent limitations [1]. Unconventional electrical machines consist of superconducting materials. They have impressive characteristics as well their very high magnetic flux density [2], the losslessness of DC, increasing the torque density [3], the weakness of loss with AC [4], and the diamagnetic perfectness when the bulk materials are used.

Numerous applications can benefit from this advanced technology, e.g., naval propulsion [5], wind power generation [6,7], electric vehicles [8], and aviation systems [9,10,11]. After the discovery of low temperature superconducting (LTS) materials, many superconducting machines have appeared. However, the machines’ cooling systems were very expensive, which led to manufacture limitations. The appearance of high-temperature superconducting materials (HTS) in 1986, gave a great boost to superconducting machines. Furthermore, several topologies were developed. Significant contributions of research works related to the field of superconducting machines, can be found in recent experimental works [12]. The authors of this work are interested in studying superconducting machines with magnetic flux concentration. 

Masson [13,14] has studied and realized a superconducting inductor made of two coaxial coils of NbTi, wherein the two coils are fed by opposite currents, and four magnetic screens are placed between them. This structure makes it possible to obtain a variation of the magnetic flux induction; its value decreases on the superconducting screens and increases between them. 

Ailam et al. [15], have successfully completed the model by adding a conventional rotating armature. The study and optimization are essentially based on the Monte Carlo method. Other studies were conducted to adapt this prototype in the aeronautics field. Moulin et al. [16,17] provided a contribution to get a prototype with bigger size. However, as the market does not meet their needs, they used a triple structure (multistack inductor). Thus, the power has been increased to 66% compared to the single superconducting inductor. El Hassan et al. [18,19] installed a circular screen bulk between the two solenoids in a way to be in an inclined position. This prototype resembles the engine of the claw pole in its operating principle. They got an induction variation of the radial magnetic flux induction approximately sinusoidal. Masson designed in [20], a superconducting machine with axial flux, it is based on the flux magnetic concentration principle. The structure is different compared to the previous ones, i.e., the superconducting coil is located outside and its armature is inside. Masson’s research team studied this model to develop future aircraft engines. 

Arish et al. [21] used the HTS-Bulk YBaCuO (YBCO) and proposed a flux-switching machine and make a comparison with the classic model. The results of this axial flux permanent magnet machine provide a visible reduction in the leakage flux. The magnetic saturation and the total harmonic distribution (THD) decrease the saturation of the machine. Douine et al. [22] used HTS bulks in electrical applications and highlighted the issue of the large sizing. This research develops three main areas, presents the YBCO bulks, a motor application which uses magnetic field trapping and shielding is developed and the HTS bulks characterized. This study should provide useful to engineers seeking to design better superconducting motors.

Namburi et al. [23] highlighted the importance of recycling the “failed” YBCO materials. The paper contains the properties of the recycled samples compared with the primary grown ones. The observed changes are related to the porosity and Y_2_BaCuO_5_ distribution. The recycled samples allow one to increase their tensile strength, hardness and flexural strength. Good correlations were identified between the recycled samples and the primary grown ones. Zhang et al. [24] presented the AC loss from the perspective of quantitative and qualitative analysis methods and the techniques used on superconductors for reduction of AC loss in the electrical machines. The paper may be used to deepen the better understanding of the AC loss and is a base guide for future research. Muralidharet al. [25] presented a production process through cold-top-seeded infiltration of bulk single grain superconductors. They investigated the properties of the bulk after the addition of CeO_2_ and Pt. Multiple methods were tested and analyzed in the microstructures. The identified characteristics were related to its magnetic properties and the high spatial homogeneity. 

Sumption et al. [26] analyzed multiple types of electrical conductors in superconducting and normal state at cryogenic temperatures. The results were compared with superconducting YBCO and MgB_2_. Two analysis cases were developed, and the results determined the filament size and offer a wide pallet of options. Polichetti et al. [27] varied the temperature in order to analyze type II superconductors. The S(H) curves were considered and the results were compared with the ones identified in the specialty literature. The relation identified between the SMP phenomenon in Jc(H) and the S(H) shows the availability for both of them to be attributed to a sequential crossover, regardless of the type II superconductor, which is between a less effective pinning to a more effective pinning.

Sizochenko and Hofmann [28] investigated the relations between the basic physicochemical properties and the superconductive inorganic materials at critical temperatures from a quantitative perspective. The paper provides a way to maintain a generalizable model while making more accurate interpretations. Li et al. [29] applied active vibration control to two linear electromagnetic actuators on each bogie. The results of the experiments showed that the used control strategies are comparable with the passive bogie system and to improve the classic systems’ stability. Kutt et al. [30] worked to increase the offsetting system reactive torques and the propulsion efficiency. This concept was introduced in the design of the wind turbines. Is presented a counter-rotating field axial-flux generator and armature. The design process contains three steps: the first one is analytical calculation, the second FEM simulation and the third one is the prototype experimental measurements. 

Statra et al. [31] developed a semi-analytical approach based on the volume integral method conceived to commute torque and forces in a fully high temperature superconducting ironless axial flux machine (HTS IAFM). The multi objective optimization method and the genetic algorithm were used to minimize the superconducting wire length in the structure and to maximize the magnetic torque. Padmanathan et al. [32] reviewed the evolution of wind energy conversion system (WECS)-based permanent magnet synchronous generators (PMSGs) and compared the direct driven and geared conversion systems. The paper describes the electrical machines used in WECS classification, presents multiple PMSG topologies and design aspects, emphasizes the conceptual framework and the control for WECS. 

Most of the previous structures are superconducting radial flux machines. Our research works revolve around the superconducting machine with axial flux. According to Gieras [33], these kinds of machine have a wide field of application with good prospects due to their higher torque density and large power density [34]. In fact, they ensure low cogging torque with low noise and low magnetic leakage flux. Ailam et al. [35,36] proposed a prototype of axial flux machine, carrying out studies on superconducting inductors using the Monte Carlo stochastic method. 

**Figure 1:**
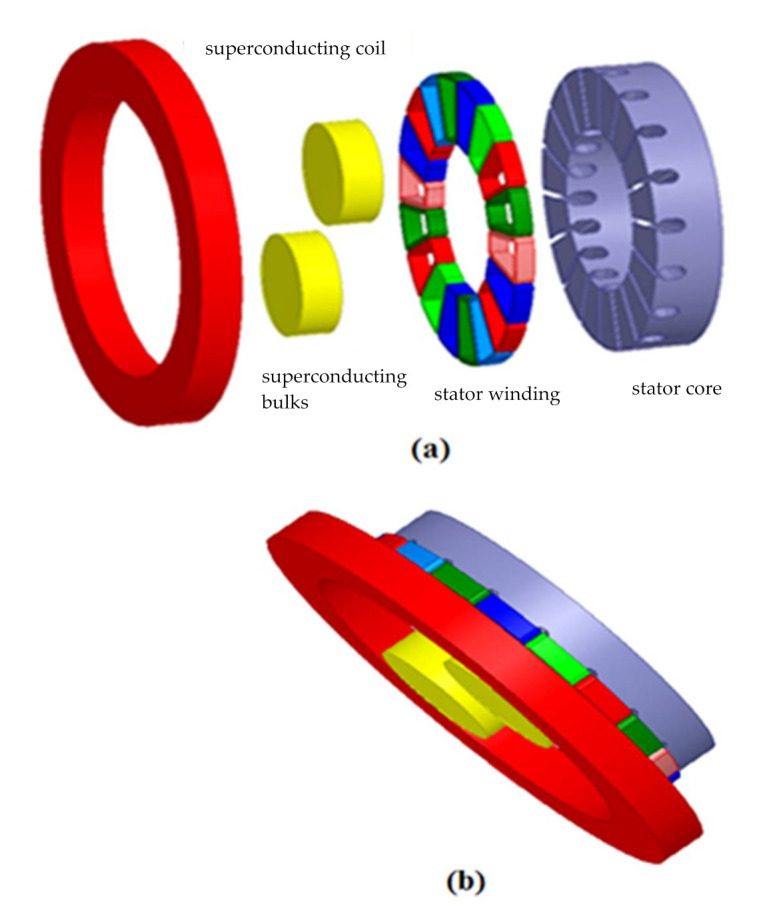
Axial flux superconducting motor configuration: (**a**) Exploded view and (**b**) Assembly view.

There are numerous ongoing efforts all around the world on increasing the power density of machines using cryo-electrification [37,38,39,40], especially for future aviation applications [41,42,43]. In this work, the authors thus decided to design, optimize and realize a new kind of superconducting machine, based on the principle of magnetic flux concentration. As a first approach, a 3D model is designed according to the previous prototype, a conventional armature is added and the finite element (FE) method is used. This improved model is illustrated in Figure 1.

The configuration presented in Figure 1 illustrates the different element of the considered prototype; it consists from left to right of a superconducting coil, two superconducting bulks of YBCO, eighteen coils of armature and stator’s core. Once the simulation and optimization parts are achieved, the realization phase starts, and it will be followed by the experimental validation.

## 2. Superconducting Inductor Design

In this section, a first technical comparison between the superconducting and the permanent magnet inductors is presented. Subsequently, the authors identified the operating point of superconductor inductor thanks to simulation results, which led to a coherent choice of inductor dimensions for the practical realization phase. The authors have then studied the superconducting coil and the superconducting inductor separately; a 3D the magnetic flux density is obtained.

### 2.1. Superconducting Inductor vs. Conventional Inductor

**Figure 2:**
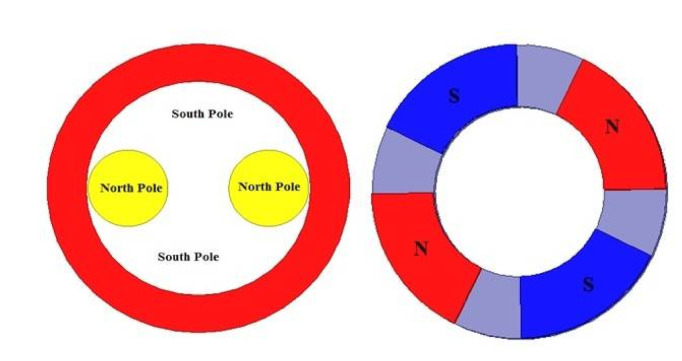
Superconducting inductor’s behaviour.

Compared to conventional machines, the proposed prototype inductor shown in Figure 2, depicts a radical change either regarding the design or the materials it consists of. 

Usually traditional machines’ inductors consist of iron, copper and magnets, for instance; the permanent magnets are set on an iron ring in the machine’s inductor. The studied inductor consists of a thick NbTi solenoid to get a strong magnetic field, which will be subsequently concentrated using two massive single crystal YBCOs. The characteristics are listed in Table 1 and Table 2. 

Compared to a permanent magnet inductor, the YBCO plates act as north poles and the space between magnetic screens behave as south poles in the superconducting inductor.

### 2.2. Operating Point of the Superconducting Inductor

To define the critical current that flows across the solenoid of the inductor, and given that the considered superconducting inductor contains a superconducting coil made of NbTi; the equation of the Ic (B) is used as follows:

J = −222.222B + 2000(1)

As an assumption, a value of 100 A/mm^2^ for the current density is considered in this simulation. This assumption leads to a DC value of 15.19 A through the solenoid.

The maximum magnetic flux induction is obtained thanks to the load line equation (maximum magnetic field on the inductor), illustrated in Equation (2):

J = 108.7B(2)

**Figure 3:**
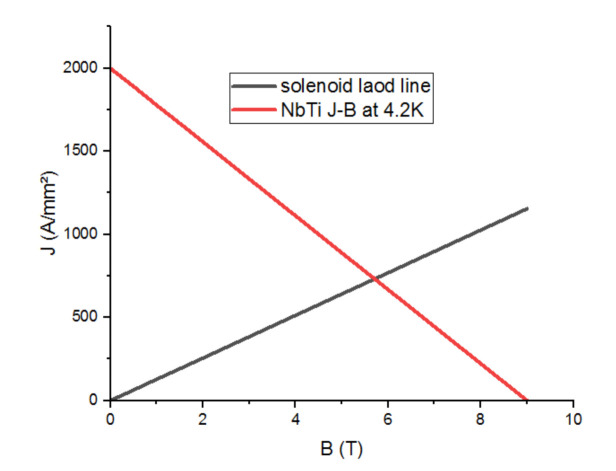
Operating point of the superconducting inductor.

The intersection between the two straight lines: the load curve and the Ic (B) curve of the low temperature material (NbTi), gives the operating point of the superconducting inductor as illustrated in Figure 3. 

It can be seen from Figure 3 that solenoid load line increases starting from a nil value to reach a value of 1300 A/mm^2^ for the current density which corresponds to the value of 12 for the magnetic flux. At a temperature of 4.2 K, the NBTi materials evolve decreasingly, starting from a value of 2000 A/mm^2^ for the current density to reach zero at a corresponding value of 9 Tesla for the magnetic flux. Thus, the critical current density reached 670 A/mm^2^ and the electrical current I = 101 A.

### 2.3. The Superconducting Inductor Design

The authors suggested 15 mm in the superconducting coil thickness, due to the availability of the corresponding YBCO plates in our laboratory. The internal radius of the superconducting coil must be enough to contain the superconducting bulks and the machine shaft, with respect to the components’ spacing. The outer radius is chosen in such a way as to obtain an induction difference of magnetic flux equal to that in the air gap of conventional machines, which is equal to 2.5 Tesla. Thus, the outer radius is 85 mm.

#### Superconducting Inductor vs. Superconducting Coil

**Figure 4:**
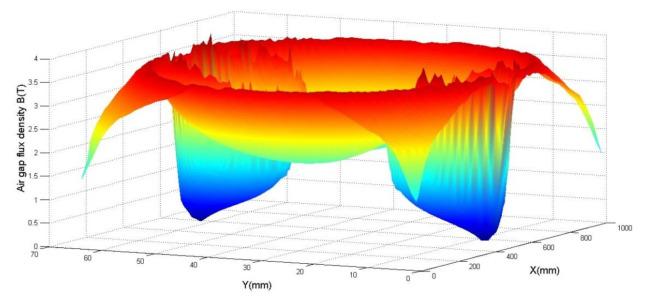
Magnetic flux density map to the inductor in 3D at a distance of 0.5 mm.

After inserting both YBCO plates in the previous structure, a magnetic flux concentration is obtained. In consequence, the magnetic field increases between the magnetic screens. The magnetic induction decreases with both superconducting bulks; a smaller value of magnetic flux induction is obtained while approaching the bulk’s center as shown in Figure 4. 

Nonetheless, the magnetic field increases at the edges of YBCO plates. We also notice that because of its lines’ focus.

**Figure 5:**
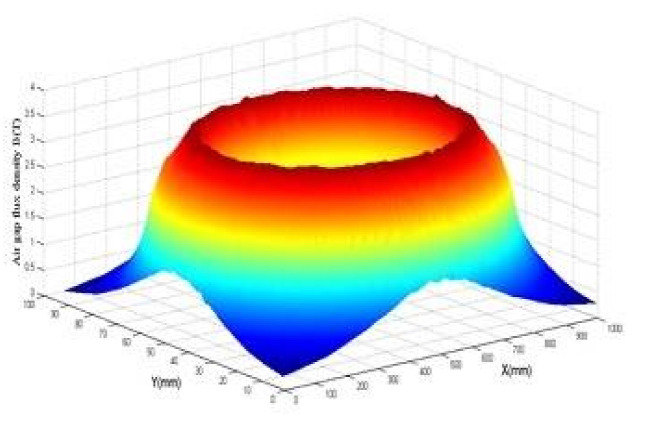
3D magnetic flux density map at 0.5 mm of superconducting solenoid.

The comparison of the inductor with the coil alone (Figure 5 illustrates the distribution of magnetic flux induction in a thick coil) reveals a higher magnetic flux induction between the superconducting bulks of the inductor.

**Figure 6:**
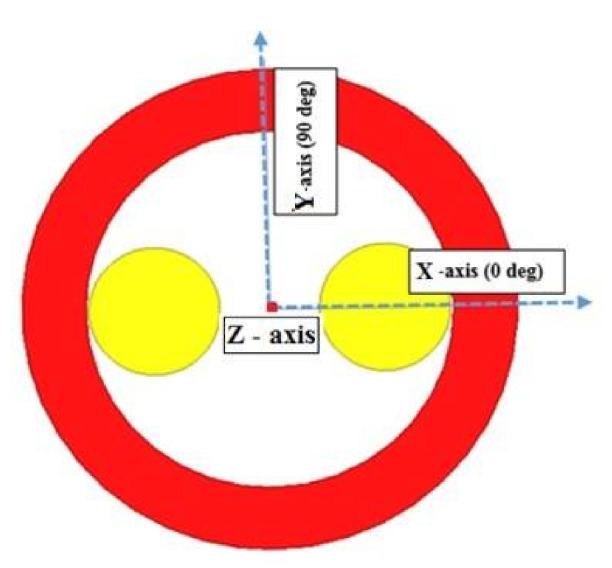
Axes to calculate magnetic flux induction.

According to Figure 6, the magnetic flux induction is calculated firstly in the YBaCuO plate for different values in the (*X*-axis) at z = 0.5 mm away from the inductor (*Z*-axis). In the second case, the magnetic flux induction is calculated at (x = 0 mm) for different values in the (*Y*-axis) for z = 0.5 mm.

**Figure 7:**
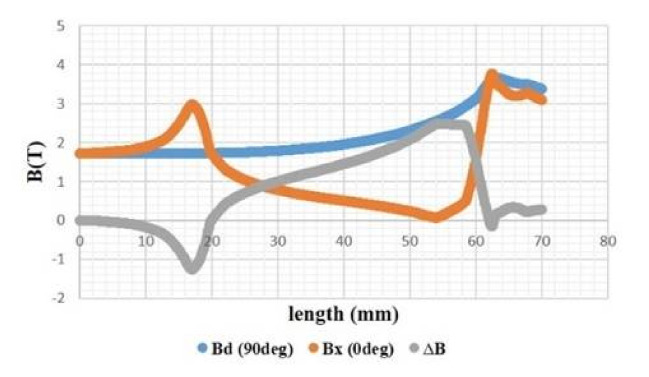
Variation of the maximum magnetic flux induction at 0.5 mm from the inductor along the radius.

After the dimensions of the superconducting inductor have been fixed (R_ext_ = 85 mm, R_int_ = 63 mm, L = 15 mm), the magnetic induction on the axis which is the pellet (Bx) and the magnetic induction on the axis which is between the two pellets (Bd) and the difference (∆B) between the two magnetic inductions (Bd-Bx) obtained, which is an important factor to obtain the electromagnetic torque in electric machines, were calculated. The magnetic flux induction calculated on *Y*-axis, Bd and Bx and ΔB are calculated, and are presented in Figure 7. 

The evolution of electromagnetic flux induction differs for each axis. A continued increase is observed in Bd (90°) to almost 4 Tesla, unlike that observed for Bx which seems to vary periodically across the radius length, it is also considered as an unstable evolution. However, ΔB reveals a difference of up to 2 Tesla in the region starting from 20 mm to 50 mm. This difference decreases to be almost after 60 mm. Hence, the electromagnetic flux induction is much stable in both directions within a radius of no less than 60 mm.

### 2.4. Prototype Optimization

The idea adopted to optimize the performance of our machine is to vary the superconducting inductor shape to get a high magnetic flux induction, and consequently the electromagnetic torque, as well as the electromagnetic power.

#### 2.4.1. Shape Optimization

**Figure 8:**
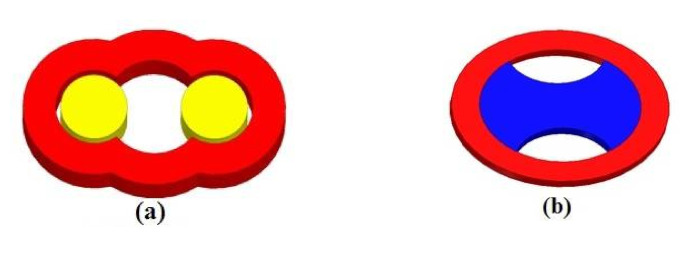
New kind of superconducting inductors: (**a**) inductor with optimized coil, (**b**) inductor with optimized YBCO plat.

To optimize the performance of our inductor, two methods can be adopted: (i) varying the superconducting coil shape while keeping the same distance between the two superconducting bulks and their length. The difference between the external and internal perimeters (22 mm) of the inductor coil as shown in Figure 8a. (ii) keeping the circular form of the inductor coil and varying the form of the YBCO bulk as shown in Figure 8b.

**Figure 9:**
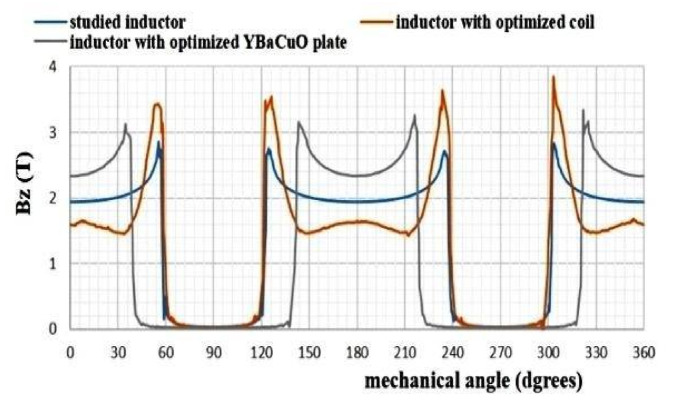
Comparison of the magnetic flux inductions created by the three superconducting inductors.

According to Figure 9, the inductor with optimized YBCO bulk presents remarkable results. In fact, it leads to get highest value of the normal magnetic flux induction as compared to the two other forms. Since this form is practically unrealizable, our study will be limited to the first form studied.

Obviously, studied inductor presents a mean value of 2 Tesla instead of 1.8 and 2.3 Tesla for inductor with optimized coil and for inductor with optimized YBaCuO plate, respectively. 

#### 2.4.2. Superconducting Wire Optimization

**Figure 10:**
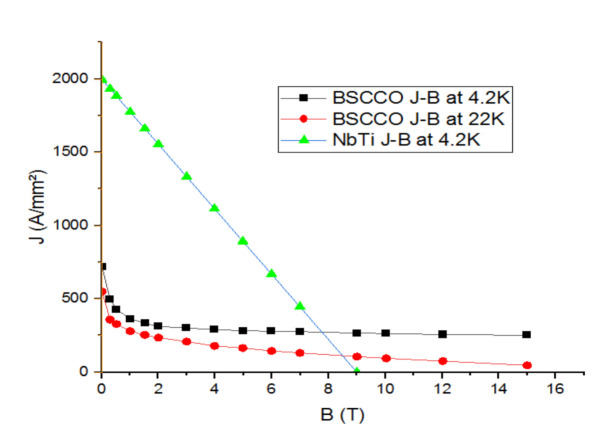
Critical currents of different superconducting wires vs. applied magnetics field.

Superconducting wires are evaluated theoretically under two different low and high temperature scenarios, i.e., LTS material at 4.2 K and HTS material at 4.2 K and at 22 K, shown in Figure 10.

It is obvious to observe that temperature affects the BSCCO J-B curves. In fact, lower electromagnetic inductor performances can be obtained by increasing the temperature.

**Figure 11:**
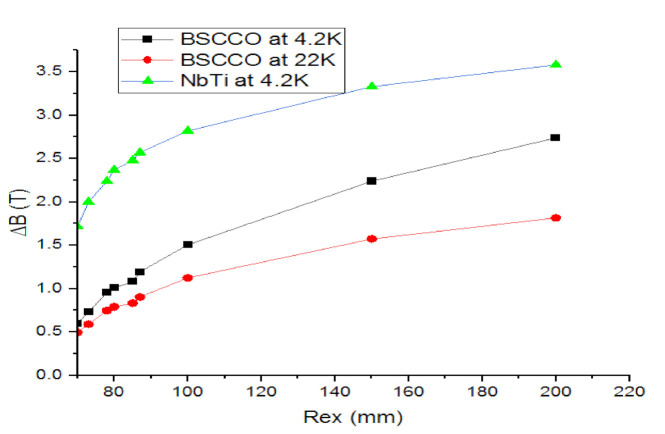
Variation maximum magnetic flux density (ΔB) vs. Rext on inductor with different wires.

Figure 11 presents the development of the maximum magnetic flux density according to outer radius which varies from 70 to 200 mm above the inductor with different wires; length and inner radius are kept to the values 15 and 63 mm, respectively. Each time the outer radius is changed the abovementioned process must be repeated. In fact, this step is useful to determine the operating point, and consequently the maximal value of the flux density on the wire. 

According to Figure 11, the inductor with LTS material at 4.2 K has a considerable maximum magnetic flux density as compared to the one with HTS material at 4.2 K and 22 K.

A proportional relation is observed with ΔB and external radius. It can be observed that the difference is more important at 4.2 K for the BSCCO. Although, the ΔB of the NbTi is greater than BSCCO at 4.2 K.

### 2.5. Superconducting Machine

The authors’ prototype contains a superconducting inductor and a three-phase conventional axial armature, as shown in Figure 1. The stator of this prototype is not concerned in this study, therefore the dimensions are chosen on the basis of Equation (3) [44,45]:

(3) $$\frac{D_{\mathrm{int}}}{D_{\mathrm{ext}}}=\frac{1}{\sqrt{3}}$$

where internal diameter (D_int_) and exterior diameter (D_ext_).

Table 3 presents the different characteristics of our machine.

**Figure 12:**
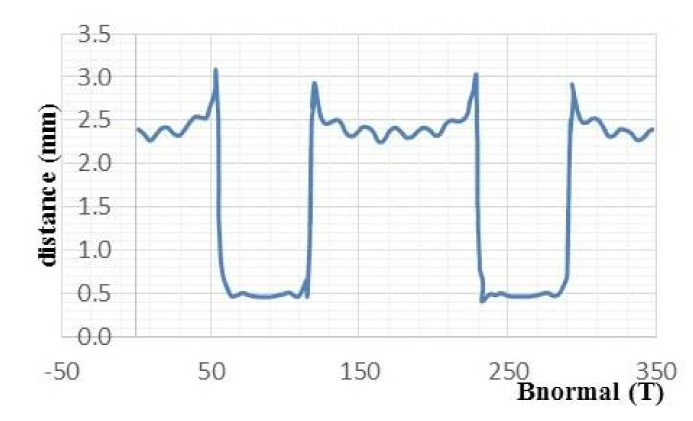
Magnetic flux induction normal in air gap created by the superconducting machine.

Figure 12 represents the magnetic flux induction normal for a 4-pole axial flux superconducting machine, whose air gap e = 0.5 mm, knowing that the inductor excitation current I = 101 A, the current density j = 670 A/mm^2^, and the rotation speed N = 1400 rpm. 

The magnetic flux induction normal reaches its maximum value between the two superconducting bulks and reaches the minimum above them.

### 2.6. Induced Voltage

**Figure 13:**
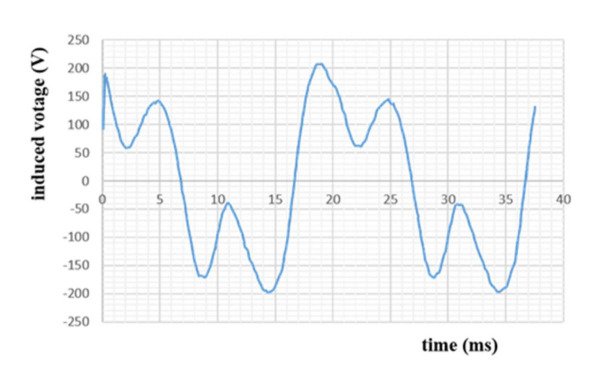
One phase of electromotive force created by the superconducting machine.

In Figure 13, the induced voltage is almost sinusoidal; it reaches the peak value of (200 V) when the stator machine is used, and it decreases when the coreless winding stator is used.

From Figure 13, It can be observed that the induced voltage swings between two maximum positive value of 200 V in a period of 20 ms. During the first 5 ms, the voltage drops to the value of 150 before reaching a negative value that mirrors the positive one.

## 3. Realization and Test

**Figure 14:**
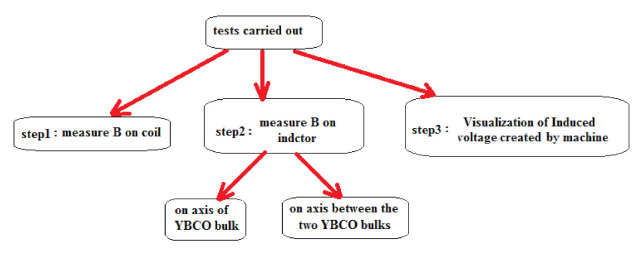
Schematic diagram of the experimental configuration.

A schematic diagram of the experimental configuration regarding the way in which the system’s connection was performed in order to perform the tests is displayed in Figure 14. For measure B, a gaussmeter (DX-102, Dexing Magnet, Jiangshu, China) was used, that is based on a Hall effect sensor, with precision and accuracy bands: a—better than 1.0% (0 ± 1000 mT), b—better than 2.0% (1000 ± 2000 mT) and c—better than 4.0% (2000 ± 3000 mT).

As a modeling method, a software program was used that is based on the finite element method, being solved simultaneously the four Maxwell equations:

(4) $$\nabla \times \;\vec{E}=-\frac{d\;\vec{B}}{\mathrm{dt}}$$

(5) $$\nabla \times \;\vec{H}=\;\vec{J}+\frac{d\;\vec{D}}{\mathrm{dt}}$$

(6) $$\nabla .\;\vec{D}=\;\rho$$

(7) $$\nabla .\;\vec{B}=0$$

Combining Equations (4)–(7) and solving them for magnetic vector potential and electric scalar potential will result in the following equation [46]:

(8) $$\nabla \times \frac{1}{\mu }\;\nabla \times \;\vec{A}+\;\sigma \;\frac{\partial \;\vec{A}}{\partial t}+\;\varepsilon \;\frac{\partial^{2}\;\vec{A}}{\partial t^{2}}=-\sigma \nabla V-\varepsilon \nabla \frac{\partial V}{\partial t}$$

The validation of the results can be summarized as follows: first, the work was started by calculated and validated B in axes of a thick coil by using both analytical and modeling methods, and then the activity was continued. In the tests phase the results were experimentally validated by FEM as shown in figures below.

### 3.1. Inductor Realized and Test

**Figure 15:**
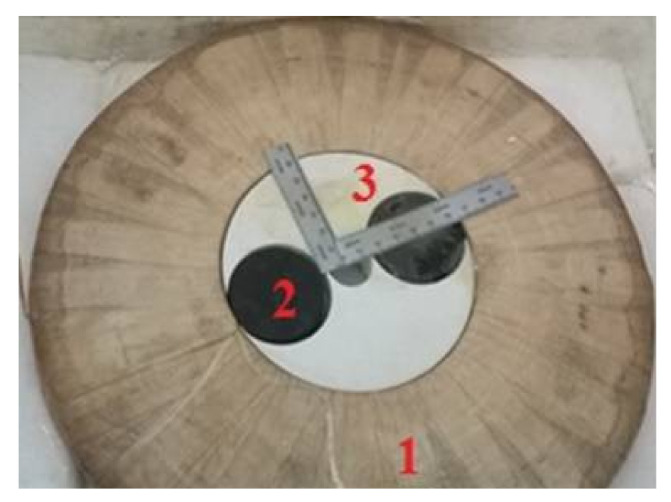
Superconducting inductor: (1) copper coil, (2) YBCO plate, (3) forex material machine.

We will introduce the prototype made (Figure 15), as well as the performed tests at the “LESI” research laboratory. To minimize the realization costs, an inductor coil of copper is realized.

The table below shows the different characteristics of this coil (refer to Table 4).

**Figure 16:**
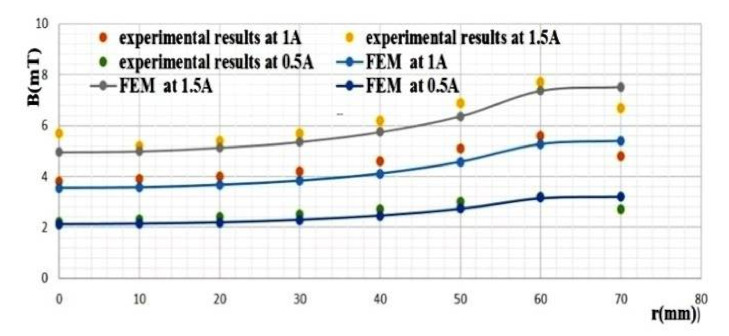
Comparative graph of the FEM vs. the experimental magnetic flux induction on copper coil.

The three lines in Figure 16 indicate the magnetic flux induction variation versus the coil radius. The magnetic flux induction increases while approaching the coil’s internal radius. 

**Figure 17:**
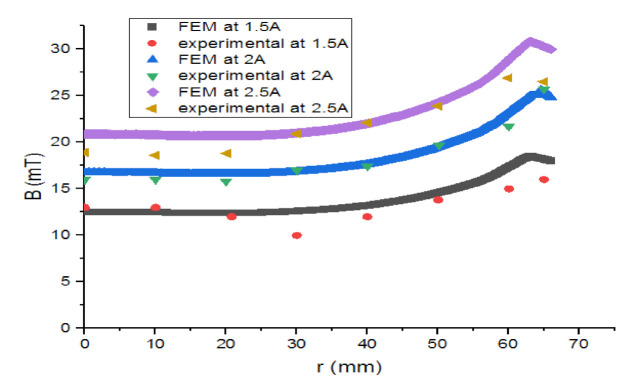
Comparative graph of the FEM vs. the experimental magnetic flux induction between the superconducting bulks.

The three lines in Figure 17 indicate the magnetic flux induction variation versus the inductor radius between the superconducting bulks. As expected, the magnetic flux induction increases while approaching the inductor’s internal radius. 

**Figure 18:**
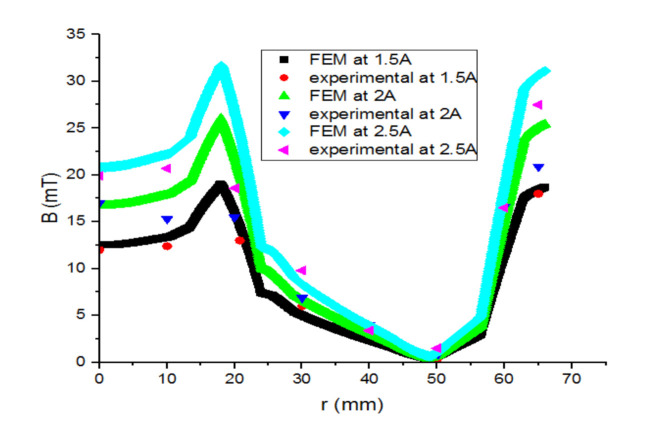
Comparative graph of the FEM vs. the experimental magnetic flux induction behind superconducting bulks.

In Figure 18 the magnetic flux induction becomes almost null on the superconducting bulk centre. The curves of Figure 16, Figure 17 and Figure 18 show that there is a concordance between the simulation results and those of the empirical part.

**Figure 19:**
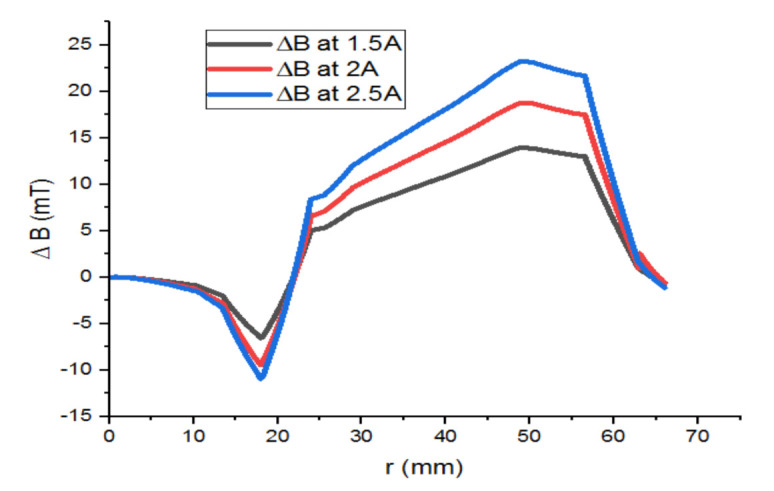
ΔB of superconducting inductor versus different currents.

Figure 19 presents the difference between the two previous magnetic flux induction graphs. It is remarkable that ΔB increases when the inductor’s current intensity is high, and vice versa. The electromagnetic torque is proportional to ΔB, as well as to the power density.

### 3.2. Realization of the Armature Winding

**Figure 20:**
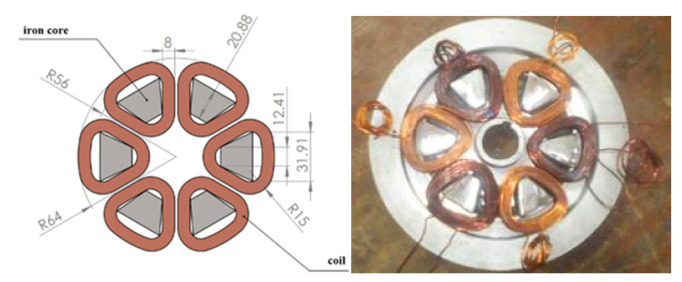
Design and realization of the stator of the machine.

Figure 20 and Table 5 present the design, dimensions, and realization of the stator of our prototype. It is a pulley which consists of six coils made of copper, All of them contain a laminated trapezoidal iron core in order to reduce the eddy current loss. 

### 3.3. Induced Electromotive Force Created by Prototype

**Figure 21:**
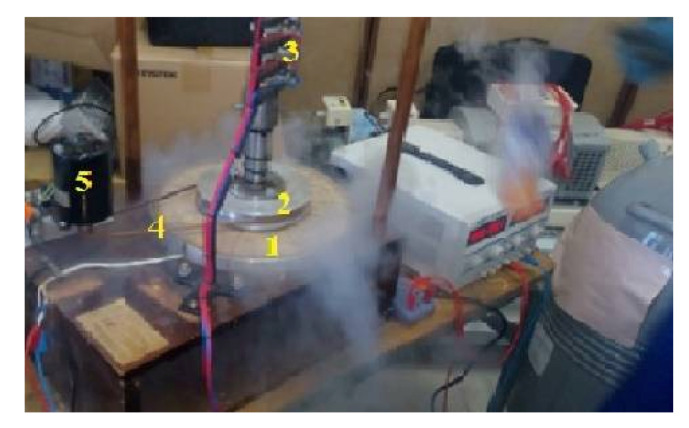
Prototype under tests: (1) inductor, (2) stator, (3) brushes and rings, (4) transmission belt, (5) synchronous motor.

To finalize the testing step, we take the model as a whole (the armature and the inductor) and test it as a generator. To optimize the operation conditions of the superconducting bulks, a cooling process is required under the effect of magnetic field (Figure 21). The inductor (coil and two YBCO bulks) is immersed in a bath of liquid nitrogen (77 k) after that it is supplied with a direct current, this method is known as zero field cooling. Therefore, the inductor coil is fed with an excitation current Iexc = 2.85 A. Afterward, liquid nitrogen is dumped on the inductor. The armature is driven by a second motor (single-phase synchronous motor), so that the rotational speed becomes 890 rpm. Finally, the output voltages are visualized using the Digital Signal Processing and Control Engineering (DSPACE) software (dSPACE Ltd., Melbourn, UK). 

**Figure 22:**
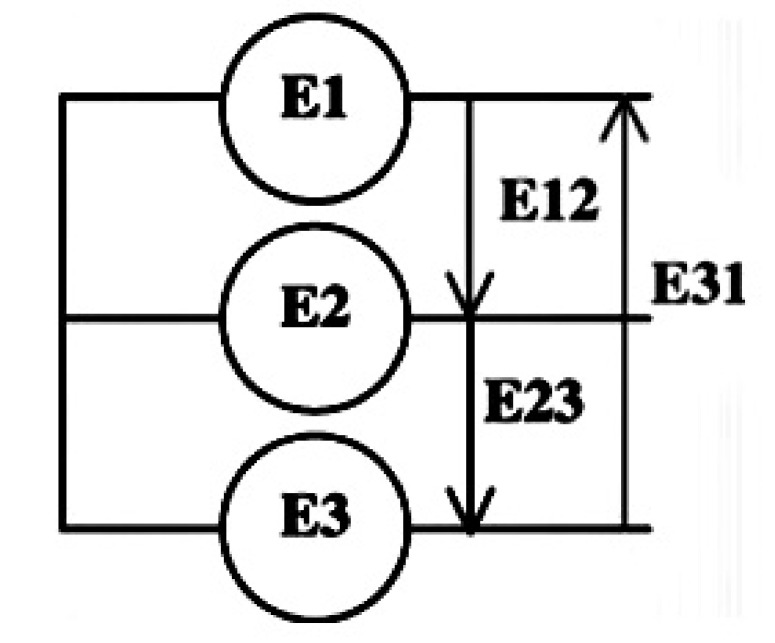
Measured voltages.

The machine is tested as a generator with an opened or short-circuited armature (no-load test) with the principle scheme presented in Figure 22. The armature ends are connected to a DSPACE to observe the line to line voltages [15].

**Figure 23:**
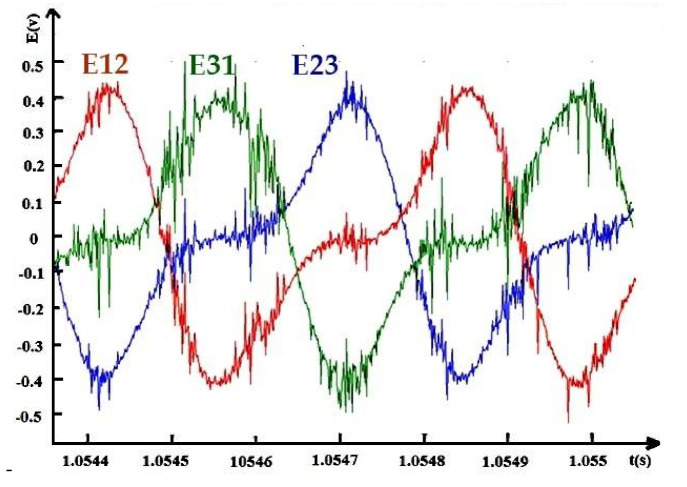
Induced voltage created by the realized machine; I_exc_ = 2.85 A, N = 890 rpm.

Results are shown in Figure 23 below. The high attractive force between the rotor and the stator creates the distortion problem. The results are satisfactory and almost sinusoidal.

The data in Figure 23 were recorded thanks to the DSPACE platform, which is why high fluctuations appeared.

## 4. Conclusions

Superconducting motors represent a promising way to make superconductivity commercially operational in real electric power systems. The fast development in the superconducting materials industry is related to the improvement of the performance of these electric motors.

A new kind of partially superconducting machine model with axial flux is designed and studied in this work. It is based on the principle of magnetic flux concentration. This unconventional inductor, which consists of two YBaCuO (YBCO) plates and the NbTi superconducting solenoid is immersed in liquid nitrogen. The superconducting coil is used for creation of the magnetic flux and it is concentrated by the YBaCuO plates. Optimization of the inductor is carried out in order to increase the torque and the power density as well.

To make the cooling process easier and more economical, this unconventional machine has a stationary superconducting inductor positioned horizontally.

The main results shown that magnetic flux is considerable between the inductor’s YBaCuO plates and it almost null behind them. Experimentally, the obtained induced voltage is almost sinusoidal, which is common to conventional machines. In perspective, a fully superconducting machine is the subject of optimization and a cooling procedure (this part is targeted as a research direction that will be addressed by the authors in the near future).

## Figures and Tables

**Table 1:** Superconducting inductor characteristics.

Characteristics	Symbol	Dimensions
External radius	Rex	85 mm
Internal radius	Rin	63 mm
Length	L	15 mm
Current density	j	670 A/mm^2^
Intensity current	i	101 A
Length of wire	L_0_	787 m
Number of turns	N	1700

**Table 2:** Superconducting materials characteristics.

**Superconducting Wire**	
Type	NbTi
Number of filaments	56
Diameter With/without insulation	0.44 mm/0.40 mm
section	0.1519 mm^2^
form	round
**Superconducting Bulks**	
Type	YBCO monocristal
Number	2
mass	158 g
Shape	disc
Radius	22.5 mm
thickness	15 mm

**Table 3:** Characteristics of the machine’s stator.

Characteristics	Symbol	Dimensions
Radius externe	Rex	62 mm
Radius interne	Rin	36 mm
Length	L	25 mm
number of slots	Ns	18
Number of coils	Nc	18
Type of winding	-	winding on the tooth

**Table 4:** Characteristics of the solenoid stator.

Characteristics	Dimensions
Radius externe	150 mm
Radius interne	63 mm
Thickness	15 mm
Number of layers	86 mm
Number of turns	1152
Material of manufacture	copper
Diameter/Wire section	1 mm/0.785 mm^2^
mass	5.5 kg

**Table 5:** Characteristic of the realized stator.

Characteristics	Dimensions
Number of phases	3
Number of coil	6
Number of turns per coil	95
wire section	0.785 mm^2^

## Data Availability

Not applicable.
